# Correlation study on different types of chronic sinusitis and eustachian tube dysfunction: analysis of prevalence and risk factors

**DOI:** 10.1016/j.clinsp.2026.100890

**Published:** 2026-02-17

**Authors:** Qihang Zhang, Qianjin Zeng, Jingtao Wang, Fu Feng, Guangke Wang

**Affiliations:** a Department of Otorhinolaryngology and Head and Neck Surgery, Zhengzhou University People's Hospital, Zhengzhou, China; b Department of Otorhinolaryngology and Head and Neck Surgery, Henan Provincial People's Hospital, Zhengzhou, China

**Keywords:** Chronic Rhinosinusitis, Functional endoscopic sinus surgery, Eustachian tube dysfunction, Eosinophilic chronic Rhinosinusitis with nasal polyps

## Abstract

**Objectives:**

This study investigated the Eustachian Tube Dysfunction (ETD) prevalence and its influencing factors in Chronic Rhinosinusitis (CRS) patients undergoing Functional Endoscopic Sinus Surgery (FESS), based on postoperative histopathological subtypes. The findings may aid in the diagnosis and individualized management of CRS with comorbid ETD.

**Method:**

This study included 92 patients who underwent FESS surgery for CRS and were hospitalized in the Otolaryngology Head and Neck Surgery Department of Zhengzhou University People's Hospital from January 2024 to May 2024. According to the postoperative pathological results, the patients were divided into the Chronic Rhinosinusitis without Nasal Polyps (CRSsNP) group and the Chronic Rhinosinusitis with Nasal Polyps (CRSwNP) group. The CRSwNP group was further divided into an Eosinophilic Chronic Rhinosinusitis with Nasal Polyps (EosCRSwNP) subgroup. General information, imaging examination data, 7-item symptom score scale for eustachian tube dysfunction (Eustachian Tube Dysfunction Questionnaire-7, ETDQ-7), and eustachian tube pressure score (Eustachian Tube Score, ETS) of all included patients were collected. The prevalence of ETD in patients with different pathological types of CRS, the influencing factors of eustachian tube function in preoperative CRS patients, and the consistency of the two eustachian tube function detection methods and the test efficacy of the two eustachian tube function detection methods were analyzed.

**Results:**

Correlations & Risk Factors: CRSwNP: Serum total IgE moderately correlated with preop ETDQ-7 (ρ = 0.497, p = 0.001). EosCRSwNP: Age (γ = 0.534, p < 0.01) and IgE (ρ = 0.506, p < 0.05) strongly correlated with ETDQ-7. IgE was an independent risk factor (OR = 1.010). Using the ETDQ-7 score and ETS score as the main diagnostic methods for ETD indicates that there is no statistically significant difference in the prevalence of ETD among the three groups of patients (CRSsNP, CRSwNP, and EosCRSwNP) (p > 0.05). Two types of examination methods were used to test the preoperative eustachian tube function of patients with different pathological types of CRS. The consistency degree of the results of the ETDQ-7 score and the ETS score was different. Among them, the patients in the EosCRSwNP group had the highest score, while those in the CRSsNP group had the lowest score (CRSsNP group: κ = 0.396, p < 0.01; CRSwNP group: κ = 0.421, p < 0.01; EosCRSwNP group: κ = 0.563, p < 0.01).

**Conclusion:**

ETD Incidence & Correlation: ETD occurrence in CRS varies slightly by subtype but remains comparable overall. CRSwNP patients show a significant association between ETD symptoms and serum total IgE levels. The ETDQ-7 score and the ETS score, as subjective and objective methods for evaluating the function of the eustachian tube, have good consistency in results and can relatively accurately assess the eustachian tube function of patients with CRS.

## Highlights

• Eustachian tube dysfunction prevalence does not differ across chronic sinusitis subtypes.
• Serum IgE levels correlate with eustachian tube dysfunction in CRSwNP.
• Total IgE and age correlate with ETD in EosCRSwNP, IgE as a risk factor.
• ET function assessment shows moderate-good consistency between ETDQ-7 and ETS results.

## Background

Chronic Rhinosinusitis (CRS) is a chronic disease characterized by inflammatory responses of the mucosa in the paranasal sinuses, with a course of more than 12-weeks[1]. According to different clinical classifications, CRS can be divided into chronic rhinosinusitis without nasal polyps (Chronic Rhinosinusitis without Nasal Polyps ‒ CRSsNP) and chronic rhinosinusitis with nasal polyps (Chronic Rhinosinusitis with Nasal Polyps ‒ CRSwNP).CRSwNP is classified into eosinophilic chronic rhinosinusitis with nasal polyps (EosCRSwNP) and non-Eosinophilic Chronic Rhinosinusitis with Nasal Polyps (non-EosCRSwNP) based on different clinical manifestations and inflammatory cell infiltration in the tissues. EosCRSwNP has a high postoperative recurrence rate and poor prognosis[2,3], and has attracted extensive attention in the academic community.

**Fig. 1:**
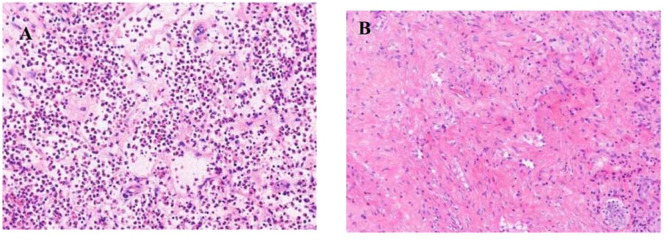
(A) EosCRSwNP Histopathological features. (B) non-EosCRSwNP Histopathological features.

**Fig. 2:**
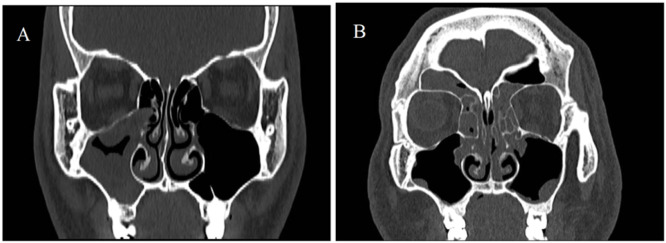
(A) CRSsNP CT imaging features. (B) RSwNP CT imaging features.

The histopathological features of EosCRSwNP are shown in Fig. 1A. The histopathological features of EosCRSwNP are shown in Fig. 1B. The CT imaging features of CRSsNP are shown in Fig. 2A. The CT imaging features of CRSsNP are shown in Fig. 2B.

The Eustachian Tube (ET) is a duct connecting the nasopharynx and the middle ear tympanic cavity, playing an important physiological role. When the Eustachian tube is dysfunctional, the pressure in the tympanic cavity is out of balance, which can lead to symptoms such as stuffiness, tinnitus, and pain in the ear[4]. Eustachian Tube Dysfunction (ETD) is one of the common otological manifestations of CRS, with a prevalence of approximately 15% to 42%[5]. Functional Endoscopic Sinus Surgery (FESS) is a minimally invasive surgical method based on endoscopic sinus technology, which can reconstruct the ventilation and drainage functions of the paranasal sinuses and has been widely used in clinical treatment. This article collects the general information of CRS patients undergoing FESS surgery and the results of different preoperative tests for the function of the eustachian tube, aiming to explore the potential factors that affect the function of the eustachian tube before the surgery. It also analyzes the prevalence of ETD in different types of CRS patients and the consistency of the test results of ETDQ-7 score and the ETS score. At the same time, based on the standard of the proportion of eosinophils among all inflammatory cells in the postoperative pathological tissues, it particularly discusses the factors affecting the function of the eustachian tube in EosCRSwNP patients. This provides a more comprehensive treatment reference for CRS patients, especially those with ETD-related symptoms.

## Methods

### Patient selection

This study selected 92 CRS patients who underwent FESS surgery in Zhengzhou University People's Hospital from January to May 2024. Among them, there were 45 male patients and 47 female patients. According to the postoperative pathological results, 50 CRSsNP patients, 42 CRSwNP patients, and 23 EosCRSwNP patients were extracted. This study has been approved by the Ethics Committee (Approval n° 2024-075-01). Inclusion criteria include: 1) Meeting the diagnostic criteria for CRS, with either nasal congestion or mucous or mucopurulent nasal discharge symptoms, and secondary symptoms including olfactory dysfunction and headache in the head and face. Patients with two or more of the above-related symptoms are diagnosed as CRS based on the above-mentioned criteria, combined with nasal endoscopy and sinus CT examinations and other laboratory indicators; 2) Willing to cooperate in filling out relevant scales and undergoing preoperative objective examinations; 3) Able to independently answer the scales, communicate with the patients before treatment, and obtain the consent of the patients and their families; 4) Able to accept follow-up at different time points after surgery, and able to fill out the postoperative follow-up scales and undergo objective examinations. Exclusion criteria include: 1) Subjects with other nasal and sinus diseases such as nasal and sinus tumors, fungal sinusitis, etc., in addition to chronic sinusitis; 2) Those with a history of ear diseases, combined with intracranial tumors, mental abnormalities, cleft lip and palate, otitis media, external auditory canal tumors or congenital nasal diseases; 3) Subjects with immunosuppressive diseases or undergoing immunosuppressive agent treatment; 4) Subjects who cannot or do not want to participate in this trial; 5) Patients with a recent history of temporomandibular arthritis or previous medical treatment, tympanic tube insertion, and eustachian tube balloon dilation surgery that affected the function of the middle ear and eustachian tube.

### Evaluation method

Collect the patient information of those who received CRS and FESS surgeries in the department, including age, gender, disease duration, preoperative nasal endoscopy and ear endoscopy examinations, and the content of total IgE in the patient's serum and the prevalence of allergic rhinitis obtained based on the report of allergen skin test and serum total IgE detection. The prevalence of asthma in the patient is obtained based on the current medical history and pulmonary function report. Preoperative nasal sinus CT examination is completed to evaluate the Lund-Mackay score to determine the lesion range. Meanwhile, according to the opinions of EPOS experts, the criterion for defining EosCRSwNP is that the count of eosinophils in each high-power field of the pathological tissue should be ≥ 10[6]. The CRSwNP group is divided into a subgroup, namely the EosCRSwNP group, and follow-up is conducted at 1-month, 3-months, and 6-months after surgery. The ETDQ-7 and SNOT-22 scales are filled out, and nasal endoscopy and ETS are re-examined.

#### ETS (Eustachian tube manometry score)

The ETS score was first proposed by Ockermann. The authors conducted the measurement of the pressure of the Eustachian tube by using Tubomanometry (SPIGGLE & THEIS Medizintechnik GmbH, Germany). Through nasal adaptors, different pressures (30 mbar, 40 mbar, 50 mbar) were applied to the nasopharynx, and the opening index of the Eustachian tube (R-value) was calculated. This index was then combined with the subjective symptom of whether a Click sound could be heard during swallowing or Valsalva action to obtain a score sheet with a total score of 10 points. A total score of ≤5 points can be considered as indicating restricted opening of the Eustachian tube function[7,8].

#### ETDQ-7 (Eustachian tube dysfunction questionnaire-7)

The ETDQ-7 scale is used to assess subjective symptoms of ETD. It consists of 7 items related to ear symptoms, each item is scored on a scale of 1 to 7, with a total score of 49. A total score of ≥ 14.5 or an average score of ≥ 2.1 can diagnose ETD, and it has been proven to have high sensitivity and specificity[9,10].

### Statistical analysis

Data analysis was conducted using SPSS 27.0 and visualization was performed with GraphPad Prism 10.1.2. Measurement data (normal distribution: one-way ANOVA; non-normal distribution: Kruskal-Wallis test), count data (Chi-Square test). Correlation analysis (Spearman/Pearson test to evaluate the relationship between ETDQ-7 and preoperative influencing factors), binary Logistic regression was used to determine the independent influencing factors of ETDQ-7, and the Kappa test was used to evaluate the consistency between ETS and ETDQ-7. Data presentation: normal distribution (x ± s), non-normal distribution (M [P25, P75]), count data (n [%]); p < 0.05 was considered statistically significant.

## Results

A total of 92 subjects were included in this study, among whom 50 were patients with CRSsNP and 42 were patients with CRSwNP. Among them, 23 patients with EosCRSwNP were collected. Statistical analysis was conducted on the general data of the patients, and the results showed that there were statistically significant differences in the Lund-Mackay score of nasal sinus CT before surgery, whether allergic rhinitis was present, whether asthma was present, and the serum total IgE content among the three groups (p < 0.05). Age, gender, disease duration, preoperative SNOT-22 score, preoperative ETDQ-7 score, and preoperative ETS score were not statistically significant among the three groups (p > 0.05) (see Table 1).

**Table 1:** Comparison of general data and preoperative results of tympanic tube function among different groups of patients.

	**Pathological type**			**p**
	**CRSsNP** **(n *=* 50)**	**CRSwNP** **(n *=* 42)**	**EosCRSwNP** **(n *=* 23)**	
**Age (year)**	44.32 ± 15.45	51.24 ± 15.41	51.00 ± 11.09	0.051
**Gender**				0.348
Male	22 (44.0)	23 (54.8)	14 (60.9)	
Female	28 (56.0)	19 (45.2)	9 (39.1)	
**Duration of illness (month)**	12 (4.75, 66.00)	18 (6.00, 54.00)	7 (4.20, 12.00)	0.254
**Lund-Mackay score**	5.82 ± 3.74	12.48 ± 6.04	14.96 ± 5.72	<0.010^a^
**Preoperative ETDQ-7 score**	13.74 ± 5.27	12.62 ± 5.10	13.48 ± 6.58	0.612
**Preoperative SNOT-22 score**	43.52±14.27	41.90 ± 15.13	46.09 ± 18.01	0.580
**Preoperative ETS score**				
Left ear	7.08 ± 2.28	6.29 ± 2.42	6.26 ± 3.22	0.249
Right ear	6.64 ± 2.30	6.64 ± 2.52	6.09 ± 2.52	0.621
**Is there any accompanying allergic rhinitis?**				0.010^a^
Yes	23 (46.0)	25 (59.5)	15 (65.2)	
No	27 (54.0)	17 (40.5)	8 (34.8)	
**Is there any accompanying asthma?**				<0.010^a^
Yes	7 (14.0)	6 (14.3)	11 (47.8)	
No	43 (86.0)	36 (85.7)	12 (52.2)	
Serum total IgE content	174.65 (54.66, 283.99)	319.99 (232.70, 340.35)	338.873 (313.25, 344.36)	<0.010^a^

a Indicates significant differences among the three groups, p < 0.05.

### Correlation analysis of patients' age, disease duration, Lund-Mackay score, serum total IgE content and preoperative ETDQ-7 score

A correlation analysis was conducted on the age, disease duration, Lund-Mackay score, serum total IgE content and preoperative ETDQ-7 score of 50 CRSsNP patients, 42 CRSwNP patients and 23 EosCRSwNP patients. The results showed that in the CRSwNP group, the serum total IgE content was moderately correlated with the preoperative ETDQ-7 score (ρ = 0.497, p = 0.001). In the EosCRSwNP group, age, serum total IgE content and preoperative ETDQ-7 score all showed a strong correlation (age: γ = 0.534, p < 0.01; serum total IgE content: ρ = 0.506, p < 0.05). In the CRSsNP group, there was no significant correlation between age, disease duration, Lund-Mackay score, serum total IgE content and preoperative ETDQ-7 score (p > 0.05) (see Table 2).

**Table 2:** Correlation analysis of age, disease duration, Lund-Mackay score, serum total IgE content and preoperative ETDQ-7 score.

**Pathological type**	**Clinical characteristics**	**ρ/γ**	**Sig.**	**N**
CRSsNP	Age	-0.018	0.899	50
	Duration of illness	-0.102	0.481	50
	Lund-Mackay score	0.111	0.442	50
	Serum total IgE content	-0.026	0.858	50
CRSwNP	Age	0.130	0.412	42
	Duration of illness	0.149	0.345	42
	Lund-Mackay score	0.281	0.071	42
	Serum total IgE content	0.497	0.001^a^	42
EosCRSwNP	Age	0.534	0.009^a^	23
	Duration of illness	0.384	0.070	23
	Lund-Mackay score	0.001	0.998	23
	Serum total IgE content	0.506	0.014^a^	23

a Indicates significant correlation, p < 0.05.

### Comparison of preoperative ETD among patients with different pathological types of CRS

**Fig. 3:**
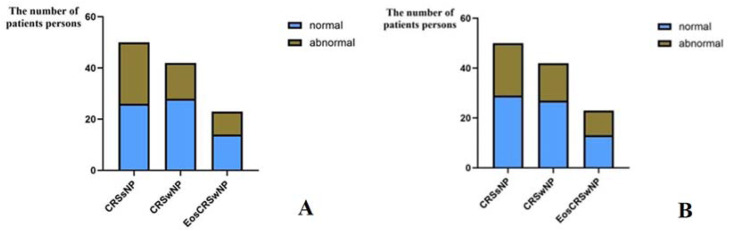
(A) ETDQ-7 score is used to detect the differences in prevalence rates among three types of patients. (B) ETS score is used to detect the differences in prevalence rates among three types of patients.

A study was conducted on the preoperative ETS scores and ETDQ-7 scores of patients with different pathological types of CRS. The criteria for diagnosing ETD were defined as ETS scores ≤ 5-points on one or both ears, and the total score of ETDQ-7 ≥ 14.5-points. The results showed that among CRSsNP patients, there were 24 patients with ETDQ-7 scores ≥ 14.5-points, with a prevalence rate of 48.0%, and 21 patients with ETS scores ≤ 5-points, with a prevalence rate of 42.0%. Among CRSwNP patients, there were 14 patients with ETDQ-7 scores ≥ 14.5-points, with a prevalence rate of 33.3%, and 15 patients with ETS scores ≤ 5-points, with a prevalence rate of 35.7%. Among EosCRSwNP patients, there were 9 patients with ETDQ-7 scores ≥ 14.5-points, with a prevalence rate of 39.1%, and 10 patients with ETS scores ≤ 5-points, with a prevalence rate of 43.5%. Horizontal comparison revealed that there was no significant difference in the prevalence rates of diagnosing ETD based on ETDQ-7 scores or ETS scores among the three groups of CRSsNP, CRSwNP, and eosinophilic CRSwNP patients (p > 0.05) (see Table 3 and Fig. 3A and 3B).

**Table 3:** Comparison of preoperative ETD among patients with different pathological types of CRS and those diagnosed by different methods.

**Diagnosis method**		**Pathological type**			**χ^2^**	**p**
		**CRSsNP**	**CRSwNP**	**EosCRSwNP**		
ETDQ-7	Normal	26 (52.0)	28 (66.7)	14 (60.9)	2.068	0.356
	Abnormal	24 (48.0)	14 (33.3)	9 (39.1)		
ETS	Normal	29 (58.0)	27 (64.3)	13 (56.5)	0.521	0.771
	Abnormal	21 (42.0)	15 (35.7)	10 (43.5)

### Comparison of ETD consistency detected by different inspection methods

Further comparison of the consistency of ETD detection by the two examination methods in patients with different pathological types of CRS showed that: in EosCRSwNP patients, the highest consistency rate of the two examination methods was 78.26%, (κ = 0.563, p < 0.01), indicating a moderate consistency in the detection results of the two methods; in CRSsNP patients, the consistency rate of the two examination methods was 70.00% (κ = 0.396, p < 0.01), indicating a relatively poor consistency in the detection results of the two methods; in CRSwNP patients, the consistency rate of the two examination methods was 73.80% (κ = 0.421, p < 0.01), indicating a moderate consistency in the detection results of the two methods (see Table 4).

**Table 4:** Comparison of the consistency test of preoperative ETS score and ETDQ-7 score in CRS patients with different pathological types.

		**ETDQ-7**			**Consistency test**		
		**Normal**	**Abnormal**	**Total**	**Consistency rate**	**Kappa**	**p**
**CRSsNP**							
ETS	Normal	20	9	29	70.00%	0.396	0.005
	Abnormal	6	15	21			
	Total	26	24	50			
**CRSwNP**							
ETS	Normal	22	5	27	73.80%	0.421	0.006
	Abnormal	6	9	15			
	Total	28	14	42			
**EosCRSwNP**							
ETS	Normal	10	3	13	78.26%	0.563	0.007
	Abnormal	2	8	10			
	Total	12	11	23			

*Indicates that the two sets of detection results are consistent, and p < 0.05.

### Multivariate Logistic regression analysis of general data and preoperative ETDQ-7 scores of patients with 3.4 CRSsNP and CRSwNP

**Fig. 4:**
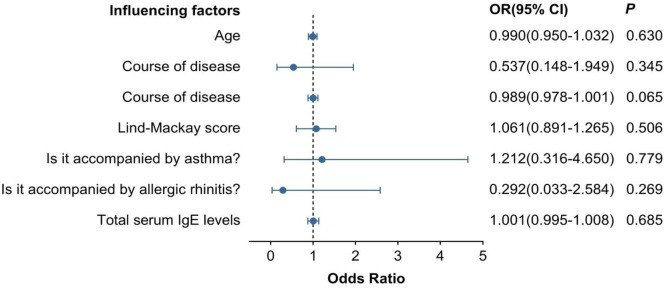
Multivariate Logistic regression analysis of general data of CRSsNP patients and preoperative ETDQ-7 scores.

**Fig. 5:**
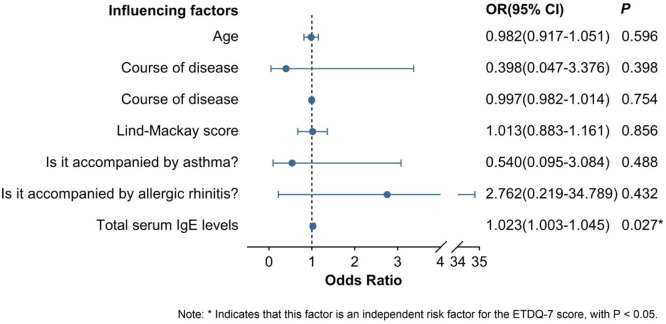
Multivariate Logistic regression analysis of general data of CRSwNP patients and preoperative ETDQ-7 scores.

ETD was diagnosed based on the total score of ETDQ-7 ≥ 14.5. After including the patient's age, gender, disease duration, Lund-Mackay score, whether accompanied by allergic rhinitis, whether accompanied by asthma, and serum total IgE content into the multivariate binary logistic regression analysis, it was shown that all the influencing factors were not independent risk factors for the ETDQ-7 score in CRSsNP patients. In CRSwNP patients, except for serum total IgE content (OR = 1.010, 95% CI: 1.000‒1.020), which was an independent risk factor for ETDQ-7 score, the rest were not independent risk factors for the patients (see Fig. 4, Fig. 5).

## Discussion

The Eustachian tube, as a passage connecting the tympanic cavity of the middle ear and the nasopharynx, plays an important physiological role. Generally, it is believed that the muscles controlling the normal opening and closing of the Eustachian tube mainly include the palatoglossus muscle and the palatopharyngeus muscle. In addition, the Eustachian tube pharyngeal muscle and the tympanic membrane muscle also play auxiliary roles and are controlled by normal physiological rhythms (such as speech, swallowing, breathing, etc.) for opening and closing. Under pathological conditions, if the Eustachian tube cannot open and close appropriately, it can lead to Eustachian Tube Dysfunction (ETD)[11]. However, there is currently a lack of a gold standard for diagnosing ETD. The ETDQ-7 score is a subjective scale tool for diagnosing ETD and has a high sensitivity and specificity[12]. It has been proven to be a good tool for diagnosing ETD. In this study, by analyzing the correlation between the preoperative ETDQ-7 score and some general data of patients with different pathological types of CRS before surgery, it was found that in the CRSsNP group, all research subjects had no correlation with the ETDQ-7 score. In the CRSwNP and EosCRSwNP groups, there was a correlation between serum total IgE content and the ETDQ-7 score (CRSwNP: ρ = 0.497, p = 0.001; EosCRSwNP: ρ = 0.506, p < 0.05). This may indicate that the serum total IgE level plays an important role in the occurrence and development of nasal polyps. However, due to the small sample size of the EosCRSwNP group, the authors only conducted a multivariate Logistic regression analysis of age, gender, disease duration, Lund-Mackay score, whether accompanied by allergic rhinitis, whether accompanied by asthma, serum total IgE content, and preoperative ETDQ-7 score in the CRSsNP and CRSwNP groups. The results showed that in the CRSwNP group, serum total IgE content was an independent risk factor for preoperative ETDQ-7 (OR = 1.023, 95% CI: 1.003‒1.045), suggesting that IgE may be one of the important factors associated with ETD in CRSwNP.

IgE is the main antibody that mediates type I hypersensitivity reactions, especially playing an important role in allergic rhinitis. When the human body is exposed to the allergenic antigen again, the allergen will bind to the sensitized IgE and cause mast cells and basophils to degranulate, releasing inflammatory mediators such as histamine and leukotrienes, thereby causing nasal mucosal edema[13] and exacerbating the inflammatory response of the nasal mucosa. In addition, relevant studies have shown that in addition to allergens, the colonization of microorganisms in the human body, such as Staphylococcus Aureus (SA), can also stimulate the formation of IgE. This locally produced IgE is closely related to type 2 inflammatory responses and is also an important factor in the formation of nasal polyps[14,15]. There are also reports indicating that the interaction of IgE and IL-17 forms a positive feedback loop to produce more IgE-mediated and aggravate local mucosal inflammatory responses, and participate in the formation of nasal polyps[16,17]. There were significant differences between the CRSsNP group and the CRSwNP group (p < 0.05). Since the nasal mucosa and the nasopharyngeal mucosa are continuous and both consist of the same common pseudostratified ciliated columnar epithelium, the decline in the eustachian tube function of patients with CRSwNP is likely caused by the inflammatory response mediated by IgE, resulting in local mucosal edema[18]. And the CT thin-layer scanning of patients with secretory otitis media by Kourtidis et al. found that CT can quantify the local mucosal edema and thickening of the affected side eustachian tube, which has certain diagnostic value for ETD. The study also believes that the occurrence of ETD may be related to the chronic inflammation of the round process of the eustachian tube and the inner wall of the eustachian tube, which leads to local mucosal thickening and then compresses the nasopharyngeal soft tissue laterally[19]. Bowles[20] et al. also found that CRS inflammation aggravation can lead to ETD, and the ear symptoms of patients are closely related to the severity of CRS. Endoscopic sinus surgery can significantly improve the ventilation and drainage of each paranasal sinus and ETD symptoms by removing the edematous mucosa and diseased tissues in the sinus and nasopharynx. The present study also showed that in the EosCRSwNP group, age was a related factor for preoperative ETDQ-7 (γ = 0.534, p < 0.01), but similar conclusions were not reached in the other two groups. The authors believe that this may be related to the fact that the age factor further aggravates the local mucosal inflammatory response and thereby affects the symptoms of the affected ear. Some studies have indicated that, in addition to inducing mucosal edema and hypersecretion of mucus in the nasopharyngeal tube ‒ leading to ventilation and drainage impairments ‒ allergenic stimuli can also promote eosinophil infiltration through Th2-type cytokines (e.g., IL-4 and IL-13), with chronic inflammation potentially impairing the ciliary function of the tube[21, 22, 23]. Surfactant Protein D (SP-D), an active protein, has been demonstrated to play a critical role in modulating Eustachian tube responses[24]. Yu and colleagues generated an SP-D gene knockout model and induced allergic respiratory inflammation by sensitizing mice through intraperitoneal injections of ovalbumin. Their study investigated the mechanism by which IgE-mediated inflammatory responses impair Eustachian tube function. They found that in the SP-D knockout mice, both inflammatory cell infiltration and IgE levels were significantly higher compared to wild-type mice. The authors propose that under normal conditions, SP-D may mitigate damage to Eustachian tube function by directly binding allergens, thereby reducing interactions with IgE. This underscores the important role of IgE in the impairment of Eustachian tube functionality[25].

Furthermore, the authors also investigated the differences in the preoperative ETD prevalence rates among the three different pathological types of patients assessed by the above two examination methods. Among them, EosCRSwNP had the highest prevalence rate (10 cases with a prevalence of 43.5%), CRSsNP (21 cases with a prevalence of 42.0%), and CRSwNP (15 cases with a prevalence of 35.7%). This roughly corresponds to the range of 15% to 42% of the prevalence rates in the relevant study by Stoikes[5]. The authors also conducted a difference analysis of the composition ratios of the number of patients with the disease among the three groups (χ^2^ = 0.521, p > 0.05), showing that the ETD prevalence rates among the three types of CRS patients are also approximately the same. The highest prevalence rate of EosCRSwNP may be related to its higher serum total IgE content[26], although this difference is not statistically significant, suggesting that there may be more and deeper reasons for CRS patients to have ETD, which still need to be studied.

At present, there is no effective gold standard method for diagnosing ETD. Combining subjective and objective examinations may be a relatively comprehensive assessment method for diagnosing ETD. Therefore, in the present study, two examination methods, ETS score and the ETDQ-7 score, were used to evaluate the changes in the function of the eustachian tube before and after surgery in patients. To compare the differences between the two methods, the authors set ETS ≤ 5-points and ETDQ-7 ≥ 14.5-points as the diagnostic criteria for ETD. The authors transformed the evaluation results of the eustachian tube function of all preoperative patients into binary variables and conducted a consistency test. The results showed that the patients in the EosCRSwNP group had the highest consistency rate (consistency rate 78.26%, κ = 0.563, p < 0.01) for both examination methods, while the consistency rate was the lowest in the CRSsNP group (consistency rate 70.00%, κ = 0.396, p < 0.01). The results indicated that the ETD associated with EosCRSwNP patients might have a single pathological mechanism. Due to the higher level of serum total IgE content and the prevalence of allergic rhinitis in the EosCRSwNP group patients, the symptoms and inflammatory response stimulation of the eustachian tube associated with them might be more closely related[27]. While the pathological mechanism of ETD associated with CRSsNP patients might be more complex and diverse.

In conclusion, although multiple preoperative factors were evaluated and several potential correlates identified, the mechanisms underlying Eustachian tube dysfunction in CRS patients with different pathological types require further in-depth research and broader discussion. Moreover, in addition to the long-recognized prolonged alleviation of nasal symptoms, FESS also plays a role in improving Eustachian tube function in CRS patients across various pathological types[28,29]. Due to the relatively complex pathophysiology of ETD and the absence of a single, reliably definitive diagnostic method, the combined approach of subjective and objective assessments ‒ specifically, the integration of the ETDQ-7 scoring method with ETS scoring ‒ has demonstrated good diagnostic consistency. However, given that its true reliability has not been validated by other relevant studies and that different testing methods exhibit varying diagnostic validity for different types of ETD[12,30], clinicians in future routine practice should select appropriate examination methods tailored to individual patient conditions and integrate multiple test results for a comprehensive evaluation.

## Statement

This study was conducted as a prospective clinical trial and adheres to the CONSORT (Consolidated Standards of Reporting Trials) guidelines. The study protocol was approved by the Ethics Committee of Zhengzhou University People's Hospital (Approval n° 2024-075-01). All participants provided written informed consent prior to enrollment.

## Authors' contributions

Qihang Zhang: Conceptualization; methodology; writing-original draft.

Qianjin Zeng: Resources; writing-review & editing.

Jingtao Wang: Investigation; formal analysis.

Fu Feng: Validation; visualization.

Guangke Wang: Supervision; funding acquisition.

## Data availability statement

The datasets generated and/or analyzed during the current study are available from the corresponding author upon reasonable request.

## Declaration of competing interest

All authors declare no competing interests.
